# LINC01535 Attenuates ccRCC Progression through Regulation of the miR-146b-5p/TRIM2 Axis and Inactivation of the PI3K/Akt Pathway

**DOI:** 10.1155/2022/2153337

**Published:** 2022-03-17

**Authors:** Zhiming Zhang, Xiaoliang Fu, Yanyao Gao, Zhiyong Nie

**Affiliations:** Department of Urology, The Second Affiliated Hospital of Air Force Medical University, Xi'an 710038, China

## Abstract

lncRNAs, a group of eukaryotic cell genome-encoded transcripts, have been demonstrated to exert a notable impact on tumorigenesis. LINC01535, belonging to the lncRNA family, was reported to have an aberrant expression in certain types of cancers and thus affect cancer progression. Nevertheless, the expression pattern and potential roles of LINC01535 in clear cell renal cell carcinoma (ccRCC) remain to be elucidated. Here, LINC01535 expression was detected in ccRCC by RT-qPCR, cell proliferation by CCK-8 assays, and invasion by transwell assays. Besides, effects of LINC01535 on *in vivo* tumor growth were investigated by xenograft tumor models. The miR-146b-5p/LINC01535/TRIM2 interaction was evaluated via luciferase reporter assays. This study showed downregulation of LINC01535 in ccRCC. Moreover, LINC01535 upregulation attenuated *in vitro* ccRCC development and hindered *in vivo* tumor growth. Furthermore, LINC01535 sponged miR-146b-5p which had a negative correlation with LINC01535, and TRIM2 was a direct target of miR-146b-5p and mediated by LINC01535. Mechanically, LINC01535/miR-146b-5p/TRIM2 axis affected ccRCC progression by mediating the PI3K/Akt signaling. All in all, our observations suggest the LINC01535/miR-146b-5p/TRIM2 axis as a crucial role in ccRCC.

## 1. Introduction

Renal cell carcinoma (RCC) is a kind of highly malignant tumor [1] and can be classified into multiple categories according to histological types [2]. In all RCC subtypes, clear cell renal cell carcinoma (ccRCC) is the most prevalent [3]. At an early stage of ccRCC, surgical resection is frequently chosen as the standard therapeutic approach [4, 5]. Unfortunately, about 20 to 30% of ccRCC patients may develop distant metastasis and suffer from a short survival rate [6, 7]. This desperate situation occurs due to ccRCC insensitivity to chemotherapy/radiotherapy or due to a lack of effective biomarkers [8]. Therefore, it is urgent to provide more effective targets for ccRCC treatment.

lncRNAs are a bunch of eukaryotic cell genome-encoded transcripts more than 200 nucleotides in length but unable to encode proteins [9]. Increasing evidence has demonstrated the notable impact of lncRNAs on cell biology [10]. Their abnormal expression has been shown to have a close association with the occurrence and development of various diseases [11–13]. Furthermore, lncRNAs function as a critical player in mediating gene expression and meanwhile play an irreplaceable role in chromosome sedimentation, transcriptional activation, and genome modification [14–16]. More importantly, a majority of lncRNAs serve as potential biomarkers and therapeutic targets during cancer development [17, 18]. LINC01535, belonging to the lncRNA family, was found having an aberrant expression in certain types of cancers and thus affects the biological processes of these cancers. For example, Fang et al. demonstrated that LINC01535 promoted proliferation and inhibited apoptosis in esophageal squamous cell cancer by activating the JAK/STAT3 pathway; Song et al. suggested that LINC01535 facilitated cervical cancer progression via targeting the miR-214/EZH2 axis [19, 20]. However, the expression pattern and potential roles of LINC01535 in ccRCC remain to be elucidated.

The study here showed that LINC01535 had a decreased expression in ccRCC. Besides, LINC01535 upregulation attenuated *in vitro* ccRCC progression and hindered *in vivo* tumor growth. Further analysis indicated the role of LINC01535, sponging miR-146b-5p to mediate TRIM2 and subsequently regulate the PI3K/Akt pathway.

## 2. Materials and Methods

### 2.1. Tissue Specimens

Approved by the Ethics Committee of the Second Affiliated Hospital of Air Force Medical University (Xi'an, China), the study was performed with informed consent signed by all patients who underwent surgery without any preoperation chemotherapy or radiotherapy. 42 pairs of ccRCC tissues and normal adjacent tissues were collected and stored at -80°C for future analysis.

### 2.2. Cell Culture

The normal renal tubular epithelial cell line HK-2 and ccRCC cell lines (Caki-1, 769-P, ACHN and 786-O), obtained from American Type Culture Collection (ATCC, Manassas, VA, USA), were cultured in RPMI-1640 medium (Sigma, St. Louis, MO, USA) containing 10% fetal bovine serum and 1% penicillin/streptomycin and then maintained with 5% CO_2_ in a humidified atmosphere at 37°C.

### 2.3. RT-qPCR

Total RNA was isolated using TRIzol kit. RT-qPCR analysis was conducted on a CFX96 Detection System with SYBR Green PCR Master Mix (Takara, Tokyo, Japan). The reaction conditions were 96°C for 1 min, 40 cycles of 96°C for 10 sec, 60°C for 5 sec, and 72°C for 30 sec. The primers were LINC01535, 5′-GGGCGGCAGGTCACTGACAC-3′ (forward) and 5′-GCCAGCAGCCGCTGGCTTAG-3′ (reverse); miR-146b-5p, 5′-TGACCCATCCTGGGCCTCAA-3′ (forward) and 5′-CCAGTGGGCAAGATGTGGGCC-3′ (reverse); TRIM2, 5′-TGGAGAAGGAAATGGGCATG-3′ (forward) and 5′-CTGCAACCACAACATGCACCA-3′ (reverse); GAPDH, 5′-CCAGCCGAGCCACATCGCTC-3′ (forward) and 5′-ATGAGCCCCAGCCTTCTCCAT-3′ (reverse); and U6, 5′-CTCGCTTCGGCAGCACA-3′ (forward) and 5′-AACGCTTCACGAATTTGCGT-3′ (reverse).

### 2.4. Western Blot

Cells were lysed using RIPA buffer (Sigma) for extraction of total protein. The BCA protein assay kit (Pierce, Rockford, IL, USA) was used to measure protein concentration. An equal amount of protein was subjected to 12% SDS-PAGE before transferring onto polyvinylidene fluoride membranes (Millipore, Billerica, MA, USA). Subsequently, the membranes were blocked with 5% nonfat milk and then incubated overnight at 4°C with primary antibodies against TRIM2, p-PI3K, PI3K, p-Akt (T308), p-Akt (S473), Akt, and GAPDH, followed by washing three times with TBST solution and probing with corresponding secondary antibodies. ECL kits were used to visualize protein bands.

### 2.5. Cell Transfection

The pcDNA3.1-LINC01535/TRIM2, miR-146b-5p mimics/inhibitors, and the shRNA targeting TRIM2 were purchased from GeneCopoeia. Lipofectamine (Invitrogen, Carlsbad, CA, USA) was used for cell transfection. After 48 h of transfection, RT-qPCR or western blot was conducted for detection of transfection efficiency.

### 2.6. Cell Proliferation Assay

At a density of 2 × 10^3^ cells/well, cells were seeded into a 96-well plate, followed by incubation for different times. Cells were then further cultured for 4 h after addition of CCK-8 reagent (Sigma). A microplate reader was used to measure the absorbance at 450 nm.

### 2.7. Cell Invasion Assay

2 × 10^5^ cells resuspended in serum-free medium were added to the Matrigel-coated upper chamber and culture medium supplemented with 10% fetal bovine serum to the lower chamber. After 24 h incubation, invaded cells were fixed and stained and their number was counted under a microscope from five random fields.

### 2.8. Luciferase Reporter Assay

The pmirGLO vectors (Promega, Madison, WI, USA) were applied for luciferase report assays. In brief, the sequence of LINC01535 containing the predicted binding site with miR-146b-5p was cloned into a pmirGLO vector to shape the reporter vector pmirGLO-LINC01535-wild type (LINC01535-WT). LINC01535-mutation pmirGLO plasmid was also constructed and named as LINC01535-MUT. TRIM2-WT and TRIM2-MUT reporter plasmids were constructed by the same method. Constructed vectors were transfected into cells with miR-146b-5p mimics or miR-NC using Lipofectamine (Invitrogen). 48 h later, the luciferase activity was measured by the Dual-Luciferase Reporter Assay System (Promega).

### 2.9. In Vivo Assay

After being purchased from Shanghai Laboratory Animal Center, male BALB/c mice were maintained with approval of the Animal Care and Use Committee of Air Force Medical University. Transfected Caki-1 cells were subjected to subcutaneous injection into nude mice. Tumor volume was measured every week and calculated using the following formula: volume (mm^3^) = length × width^2^/2. Seven weeks later, mice were euthanatized, and tumors were weighed and dissected for experimental analysis.

### 2.10. Statistical Analysis

Data were shown as means ± standard deviation (SD), analyzed by SPSS 20.0 software, and compared by Student's *t*-test or one-way ANOVA. It was considered statistically significant if *p* < 0.05.

## 3. Results

### 3.1. LINC01535 Expression Is Decreased in ccRCC

**Figure 1:**
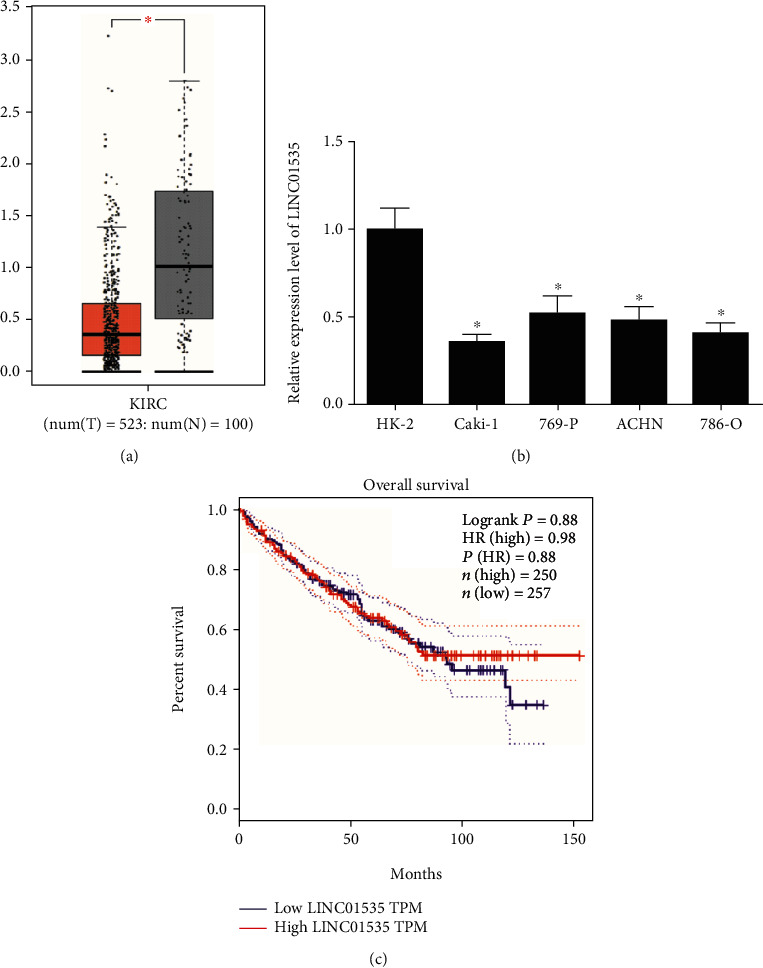
LINC01535 expression is decreased in ccRCC. (a) LINC01535 expression in ccRCC (*n* = 523) and normal (*n* = 100) samples was evaluated using the online tool GEPIA. (b) LINC01535 expression in different cell lines was examined via RT-qPCR assays. (c) The overall survival of ccRCC patients with high (*n* = 250) or low (*n* = 257) expression of LINC01535 was compared using GEPIA. ^∗^*p* < 0.05.

LINC01535 was reported to have, in certain cancers, an aberrant expression. For evaluation of LINC01535 status in ccRCC, LINC01535 expression was analyzed by the online tool GEPIA, where 523 ccRCC samples and 100 controls were included. The findings indicated a marked decrease in LINC01535 expression in ccRCC tissues compared to the normal tissues (Figure 1(a)). For confirmation of the decrease in LINC01535 expression in ccRCC, several ccRCC cell lines were used for RT-qPCR assays which suggested a lower mRNA expression of LINC01535 in ccRCC cell lines than in the control cell line (Figure 1(b)). Furthermore, there was no statistical difference between LINC01535 expression level and ccRCC patient prognosis (Figure 1(c)).

### 3.2. Upregulation of LINC01535 Attenuates ccRCC Progression

**Figure 2:**
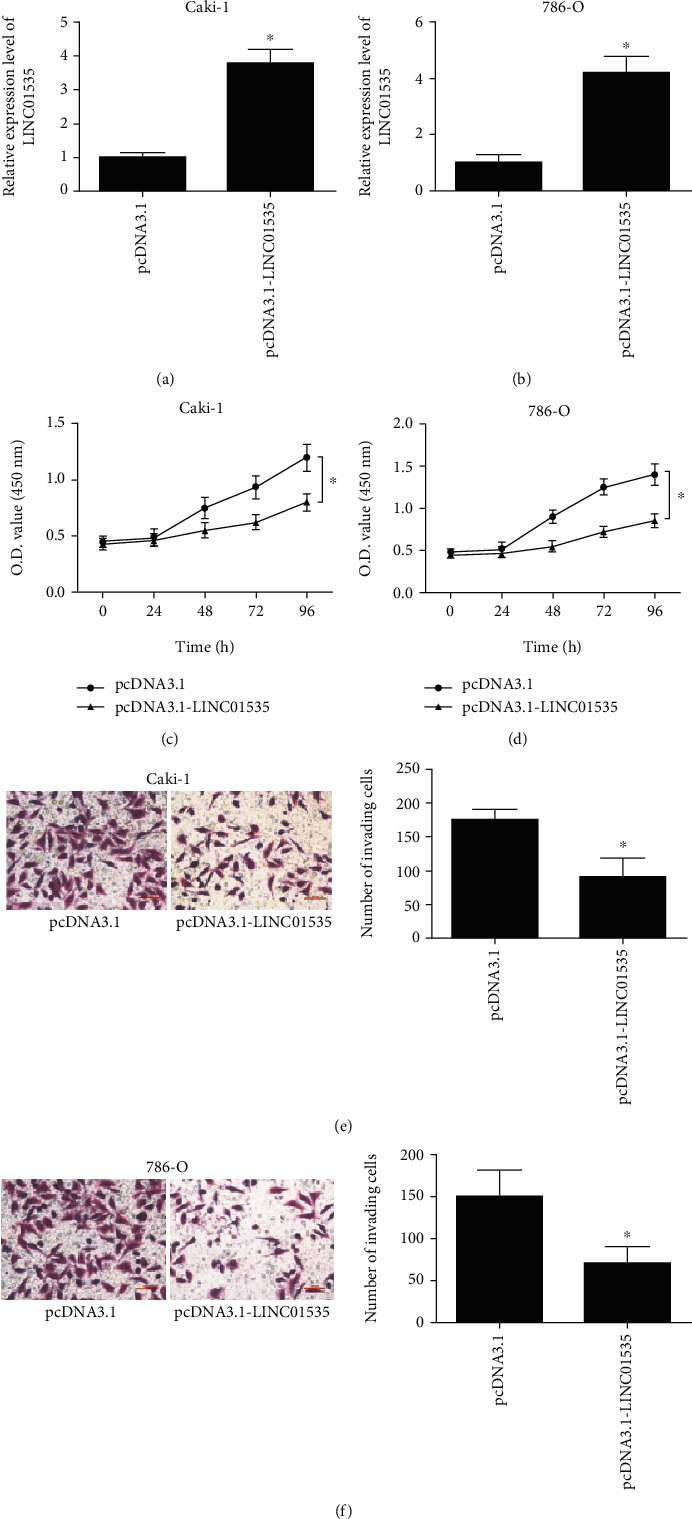
Upregulation of LINC01535 attenuates ccRCC progression. After transfection with pcDNA3.1-LINC01535 or empty vector, LINC01535 expression was measured in ccRCC cells (a, b), followed by detection of proliferation (c, d) by CCK-8 assays and invasion (e, f) by transwell assays. ^∗^*p* < 0.05.

Before exploration of the biological functions of LINC01535 in ccRCC, LINC01535 was overexpressed in ccRCC cells via transfection of pcDNA3.1-LINC01535 vectors, followed by validation using RT-qPCR assays (Figures 2(a) and 2(b)). Next, cell proliferation and invasion were detected by corresponding assays which revealed that LINC01535 upregulation significantly decreased Caki-1 and 786-O cell proliferation and invasion compared to corresponding control cells (Figures 2(c)–2(f)).

### 3.3. LINC01535 Acts as a Sponge for miR-146b-5p

**Figure 3:**
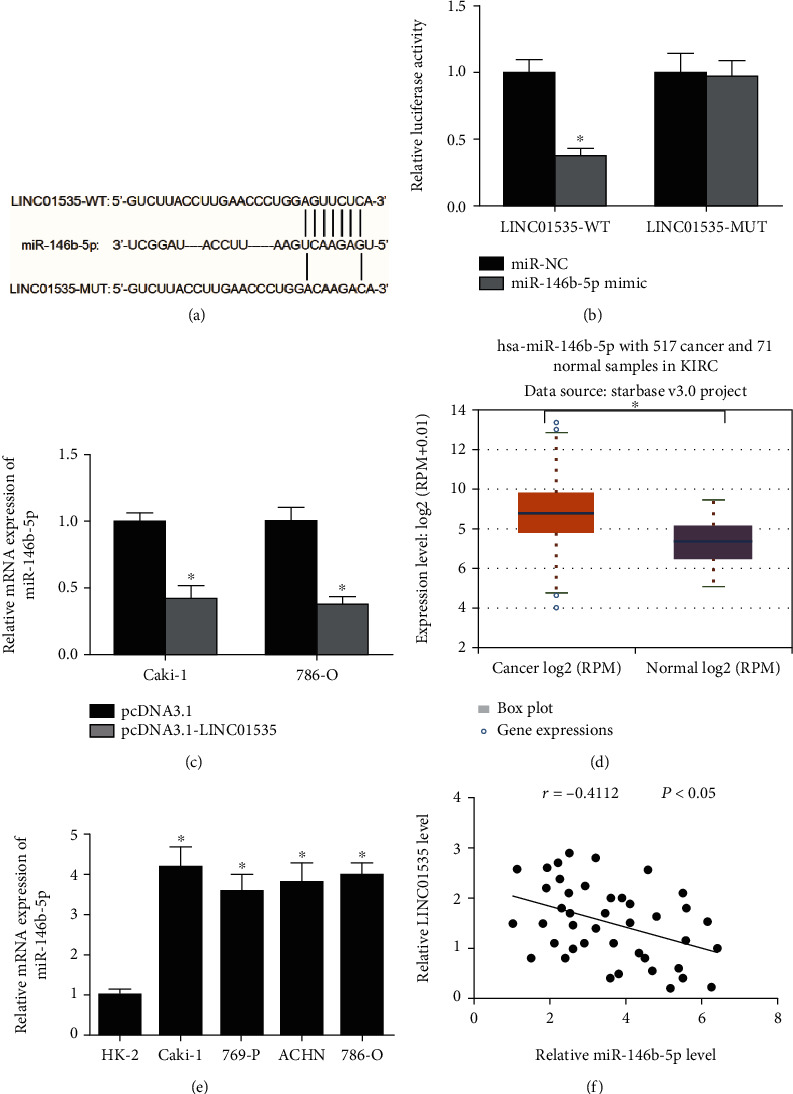
LINC01535 acts as a sponge for miR-146b-5p. (a) Potential binding sites between LINC01535 and miR-146b-5p. (b) Luciferase reporter assays verified the direct binding between LINC01535 and miR-146b-5p. (c) miR-146b-5p expression was measured in ccRCC cells after LINC01535 transfection. (d) miR-146b-5p expression in ccRCC (*n* = 517) and normal (*n* = 71) tissues was analyzed by the starBase Pan-Cancer Analysis Platform. (e) Evaluation of miR-146b-5p expression in different cell lines via RT-qPCR assays. (f) The correlation between miR-146b-5p and LINC01535 expression in ccRCC tissues. ^∗^*p* < 0.05.

Increasing evidence has shown that lncRNAs could sponge for miRNAs to mediate gene expression at a posttranscriptional manner [21]. Therefore, we investigated whether LINC01535 affected ccRCC progression in this way. Through bioinformatics analysis, it was obtained that LINC01535 was a putative target of miR-146b-5p. To verify the prediction, luciferase reporter assays were applied and these assays suggested a striking reduction in the luciferase activity of LINC01535-WT by miR-146b-5p mimics (Figures 3(a) and 3(b)).

For a further exploration of the LINC01535-miR-146b-5p interaction, LINC01535-upregulated ccRCC cells were assessed in regard to miR-146b-5p expression. As displayed in Figure 3(c), LINC01535 overexpression markedly reduced miR-146b-5p levels in ccRCC cells. Additionally, miR-146b-5p expression in ccRCC (*n* = 517) and normal (*n* = 71) tissues was analyzed by the starBase Pan-Cancer Analysis Platform. The analysis suggested an elevated expression of miR-146b-5p in ccRCC tissues compared to the control tissues (Figure 3(d)). In the meantime, evaluation of miR-146b-5p expression in ccRCC cell lines was conducted by RT-qPCR assays which revealed a much higher expression of miR-146b-5p in ccRCC cell lines than in the control cell line (Figure 3(e)). Besides, the Pearson correlation analysis was conducted, showing in ccRCC tissues a negative correlation of LINC01535 expression with miR-146b-5p expression (Figure 3(f)).

### 3.4. TRIM2 Is Targeted by miR-146b-5p and Mediated by LINC01535

**Figure 4:**
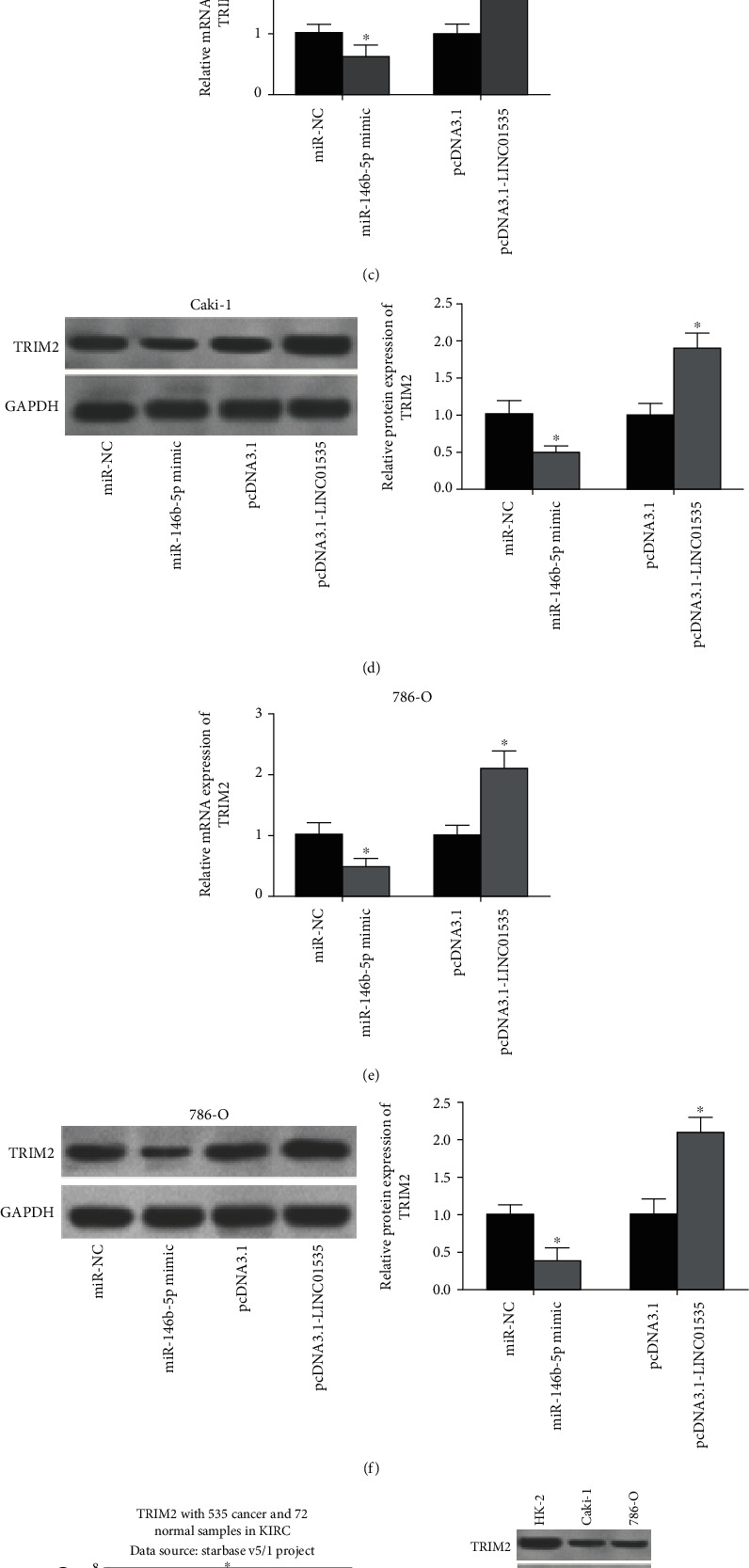
TRIM2 is targeted by miR-146b-5p and mediated by LINC01535. (a) Potential binding sites between TRIM2 and miR-146b-5p. (b) Luciferase reporter assays verified the direct binding between TRIM2 and miR-146b-5p. (c–f) TRIM2 expression was detected in ccRCC cells after miR-146b-5p or LINC01535 transfection. (g) Analysis of TRIM2 expression in ccRCC (*n* = 535) and normal (*n* = 72) tissues by the starBase Pan-Cancer Analysis Platform. (h) Detection of TRIM2 expression in different cell lines by western blot. ^∗^*p* < 0.05.

TRIM2 was a potential target of miR-146b-5p as shown by bioinformatics analysis (Figure 4(a)). Luciferase reporter assays confirmed this prediction and indicated that miR-146b-5p mimics resulted in a sharp decrease in the luciferase activity of TRIM2-WT without affecting that of TRIM2-MUT (Figure 4(b)). To make clear the relationship among LINC01535, miR-146b-5p, and TRIM2, ccRCC cells were transfected with LINC01535 or miR-146b-5p. The findings displayed that miR-146b-5p mimics decreased while LINC01535 expression vectors increased TRIM2 levels in ccRCC cells (Figures 4(c)–4(f)). The analysis obtained from the starBase Pan-Cancer Analysis Platform proved a reduction of TRIM2 expression in ccRCC (*n* = 535) tissues compared to the normal (*n* = 72) tissues (Figure 4(g)). Additionally, we obtained consistent results in ccRCC cell lines (Figure 4(h)).

### 3.5. LINC01535 Inhibits ccRCC Progression via Regulation of the miR-146b-5p/TRIM2 Axis

**Figure 5:**
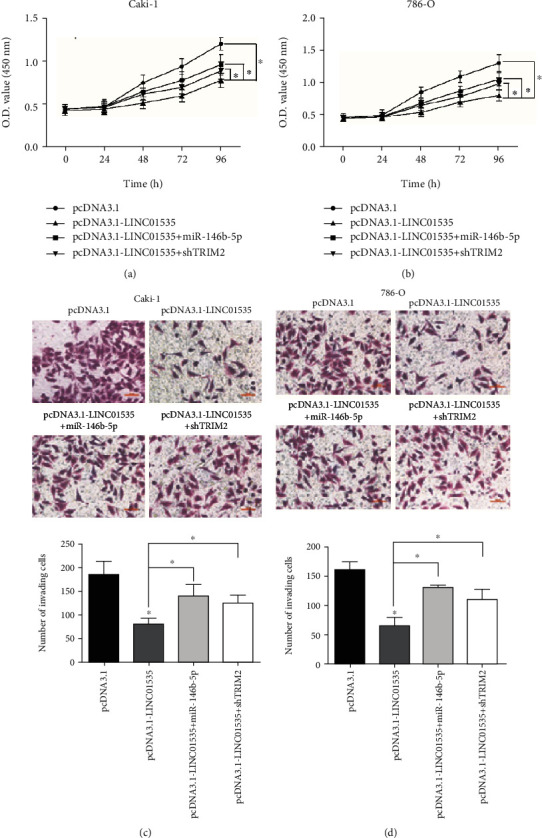
LINC01535 inhibits ccRCC progression via regulation of the miR-146b-5p/TRIM2 axis. Caki-1 and 786-O cells were cotransfected with pcDNA3.1-LINC01535 and miR-146b-5p mimics or TRIM2 shRNA, followed by detection of proliferation (a, b) by CCK-8 assays and invasion (c, d) by transwell assays. ^∗^*p* < 0.05.

For a further investigation on the role of LINC01535-miR-146b-5p-TRIM2 axis in ccRCC, rescue assays were performed in ccRCC cells. As shown by CCK-8 assays, LINC01535 inhibited ccRCC cell proliferation, which was partially reversed by miR-146b-5p upregulation or TRIM2 downregulation (Figures 5(a) and 5(b)). Similarly, transwell assays proved that Caki-1 and 786-O cell invasion was suppressed after LINC01535 overexpression but reconsolidated by miR-146b-5p mimics or TRIM2 shRNA (Figures 5(c) and 5(d)).

### 3.6. LINC01535/miR-146b-5p/TRIM2 Axis Inhibits PI3K/Akt Activation in ccRCC Progression

**Figure 6:**
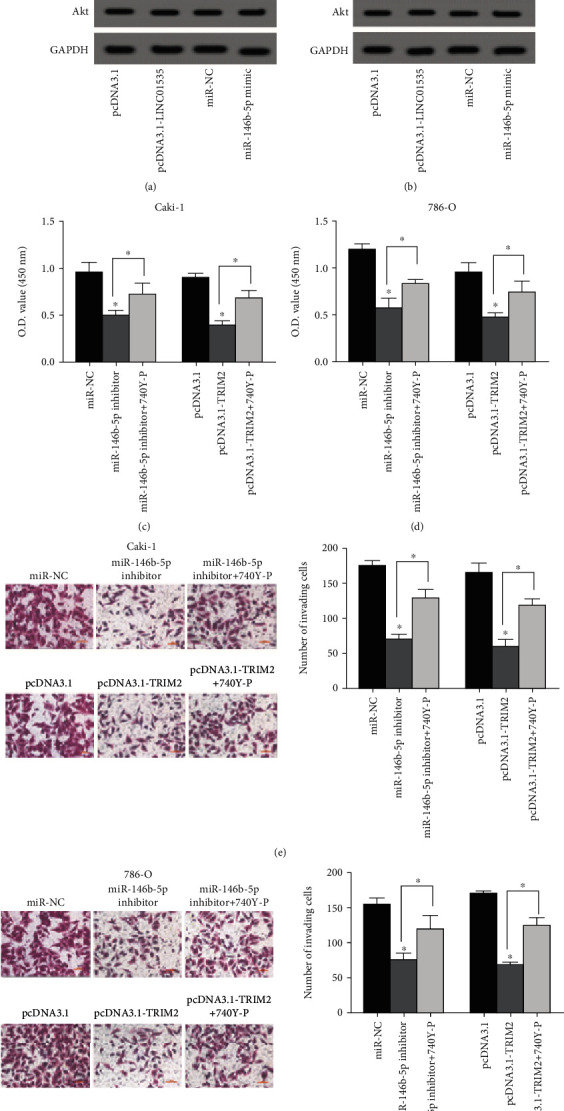
LINC01535/miR-146b-5p/TRIM2 axis inhibits PI3K/Akt activation in ccRCC progression. The protein levels of p-PI3K, PI3K, p-Akt (T308 and S473), and Akt in ccRCC cells (a, b) were measured by western blot after different treatment. Caki-1 and 786-O cells were transfected with miR-146b-5p inhibitor or pcDNA3.1-TRIM2 and then treated with 740Y-P (20 *μ*mol/L), followed by detection of proliferation (c, d) by CCK-8 assays and invasion (e, f) by transwell assays. ^∗^*p* < 0.05.

The PI3K/Akt signaling plays a crucial role in ccRCC [22, 23]. Moreover, TRIM2 has been shown to be an important regulator of the pathway [24]. Thus, effects of the LINC01535/miR-146b-5p/TRIM2 axis on p-PI3K and p-Akt (T308 and S473) were examined to explore whether the axis could exert any influence on ccRCC progression via manipulating the pathway. As shown in Figures 6(a) and 6(b), LINC01535 upregulation reduced while miR-146b-5p overexpression raised the levels of p-PI3K and p-Akt (T308 and S473) in ccRCC cells. Besides, TRIM2 expression was proved to be affected by miR-146b-5p and LINC01535. All these observations suggested a regulatory effect of the LINC01535/miR-146b-5p/TRIM2 axis on the PI3K/Akt pathway.

To make clear the implication of PI3K/Akt in ccRCC, the PI3K activator (740Y-P) was applied for further experiments. The western blot analysis exhibited a suppressive effect of LINC01535 on the levels of p-PI3K and p-Akt (T308 and S473) which was markedly attenuated by 740Y-P treatment in ccRCC cells (Supplementary Fig. 1). Then, cell proliferation and invasion were detected following 740Y-P treatment. As displayed in Figures 6(c) and 6(d), ccRCC cell proliferation was drastically weakened by miR-146b-5p inhibitor or TRIM2 upregulation, but this effect was reversed after 740Y-P treatment. Similarly, the invasive abilities of these cells were weakened by miR-146b-5p inhibitor or pcDNA3.1-TRIM2, which was abrogated by 740Y-P treatment (Figures 6(e) and 6(f)).

### 3.7. LINC01535 Inhibits ccRCC Cell Growth *In Vivo*

**Figure 7:**
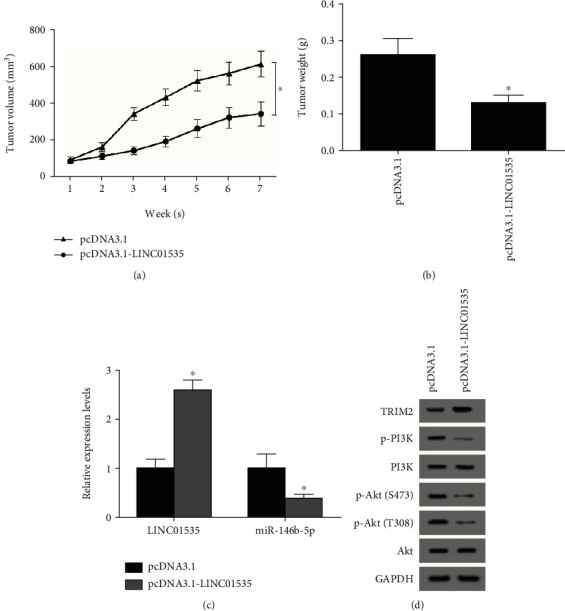
LINC01535 inhibits ccRCC cell growth *in vivo*. (a) After injection, tumor volume was measured every week. (b) Seven weeks after injection, tumors were weighed. After tumors were excised, the expression of LINC01535 and miR-146b-5p was measured by RT-qPCR (c), and the expression of TRIM2, p-PI3K, PI3K, p-Akt (T308 and S473), and Akt was measured by western blot (d). ^∗^*p* < 0.05.

For evaluation of the *in vivo* impact of LINC01535, nude mice were injected with pcDNA3.1-LINC01535-transfected Caki-1 cells. After injection, tumor volume was measured every week. As displayed in Figure 7(a), the pcDNA3.1-LINC01535 group exhibited smaller tumors than the control group. Seven weeks later, tumors were stripped and weighed, obtaining lighter tumors of the experimental group than the control group. Furthermore, in the excised tumors, the levels of related genes were determined by RT-qPCR or western blot assays which indicated an increased expression of LINC01535 and TRIM2 and a decreased expression of miR-146b-5p, p-PI3K and p-Akt (T308 and S473) in the pcDNA3.1-LINC01535 group compared to control groups (Figures 7(c) and 7(d)).

## 4. Discussion

With an increasing incidence and mortality year by year, ccRCC has attracted more and more global attention as a public health problem [25]. Among all histological types of RCC, ccRCC is most common and occupies an extremely high proportion of RCC cases [3]. The disease is characterized by a high metastasis and relapse rate, and thus, ccRCC patients frequently suffer from a poor prognosis even after partial or radical nephrectomy [6, 7]. Therefore, identification of novel biomarkers holds a great promise in ccRCC management.

Over the last decade, lncRNAs have become the research focus for their extensive capabilities in a variety of biological processes especially in tumorigenesis [10, 17, 18]. For example, lncRNA NEF, with a low expression in breast cancer, was reported to be related to poor prognosis of breast cancer patients [26]. lncRNA ANRIL was shown to have a high expression in hepatocellular cancer, and ANRIL downregulation hindered cell viability and colony formation [27]. Here, we observed a novel lncRNA transcript, LINC01535, which has been reported in many studies on cancers. For instance, LINC01535 was demonstrated to exert a pregnant regulation in osteosarcoma development [28]. Additionally, in ovarian cancer, LINC01535 expression was related to patients' poor prognosis, which provided a new strategy for therapeutic intervention of ovarian cancer [29]. LINC01535 was also observed to be aberrantly expressed in colorectal cancer and subsequently affected the cancer progression [30]. Our results indicated that LINC01535 expression was low in ccRCC. Besides, the survival analysis showed a close correlation of LINC01535 with shorter survival time of ccRCC patients. These findings demonstrated that LINC01535 may be considered a promising biomarker for judging ccRCC prognosis. Moreover, we performed a series of functional experiments which suggested that LINC01535 overexpression attenuated *in vitro* ccRCC development and hindered *in vivo* tumor growth. Contrary to our data, Fang et al. demonstrated in the esophageal squamous cell cancer a high expression of LINC01535 whose knockdown decreased proliferative but increased apoptotic rate of esophageal squamous cell cancer cells [19]. Like Fang et al., Song *et al*. reported an enhancing effect of LINC01535 on cervical cancer development [20]. The observations mentioned above suggested the contradictory role of LINC01535, tumor suppressor or promoter, in different types of cancers.

Numerous studies showed the lncRNA-miRNA-mRNA network as an important regulatory mechanism in cancer progression, where lncRNAs compete with miRNAs for binding of target mRNAs [31, 32]. A majority of lncRNAs have been confirmed to function in this way [33–35]. Here, miR-146b-5p was identified as a potential target of LINC01535 using bioinformatics tools. Many reports have provided the evidence in support of a connection of miR-146b-5p with tumorigenesis [36, 37]. For instance, Zhu *et al*. suggested dysregulation of miR-146b-5p in colorectal cancer and its enhancing effect on colorectal cancer progression [38]. Similarly, our study showed overexpression of miR-146b-5p in ccRCC as well as its oncogenic role in ccRCC progression. In addition, we found TRIM2 being targeted by miR-146b-5p. Many studies have reported the role of TRIM2 as a tumor promoter during progression of a majority of cancers such as osteosarcoma and breast cancer [24, 39]. But our study obtained contrary results that TRIM2 was downregulated in ccRCC and showed a suppressive effect on ccRCC development. All in all, our findings demonstrated the essential part of the LINC01535/miR-146b-5p/TRIM2 axis in ccRCC. Further investigation on the molecular mechanisms proved the regulatory role of the LINC01535/miR-146b-5p/TRIM2 axis in the activity of the PI3K/Akt pathway. Increasing evidence has revealed a frequent alteration of the PI3K pathway in human cancers and its key role as an important driver of tumor initiation and progression [22, 23]. Our results showed that LINC01535 upregulation decreased while miR-146b-5p overexpression increased the levels of p-PI3K and p-Akt (T308 and S473) in ccRCC cells. To better understand the involvement of the PI3K/Akt pathway in ccRCC progression, we used PI3K activator (740Y-P) for further experiments. As expected, 740Y-P treatment blocked the repressive effect of miR-146b-5p inhibitor or TRIM2 upregulation on ccRCC proliferation and invasion.

In conclusion, it was revealed here that LINC01535 expression was decreased in ccRCC. LINC01535 upregulation attenuated *in vitro* ccRCC development and hindered *in vivo* tumor growth. Furthermore, LINC01535 acted as a sponge for miR-146b-5p and had a negative correlation with it. We also validated that TRIM2 was targeted by miR-146b-5p and meantime mediated by LINC01535. Mechanically, the LINC01535/miR-146b-5p/TRIM2 axis affected ccRCC progression through regulation of the PI3K/Akt pathway. Taken together, our observations suggest that the LINC01535/miR-146b-5p/TRIM2 axis has a key role in ccRCC.

## Data Availability

The datasets used during the present study are available from the corresponding author upon reasonable request.

## Supplementary Materials

Supplementary Materials Supplementary Fig. 1: PI3K agonist 740Y-P reverses the inhibitory effect of LINC01535 on the PI3K/Akt pathway in ccRCC cells. (A, B) The protein expression of p-PI3K, PI3K, p-Akt (T308 and S473), and Akt in ccRCC cells was measured by western blot following treatment with 740Y-P (20 *μ*mol/L). ^∗^*p* < 0.05.
