# Supplementary figures and images for: LINC01535 Attenuates ccRCC Progression through Regulation of the miR-146b-5p/TRIM2 Axis and Inactivation of the PI3K/Akt Pathway
Source: J Oncol. 2022 Mar 17;2022:2153337. doi: 10.1155/2022/2153337 (PMC8947867; doi:10.1155/2022/2153337)

**A****Caki-1**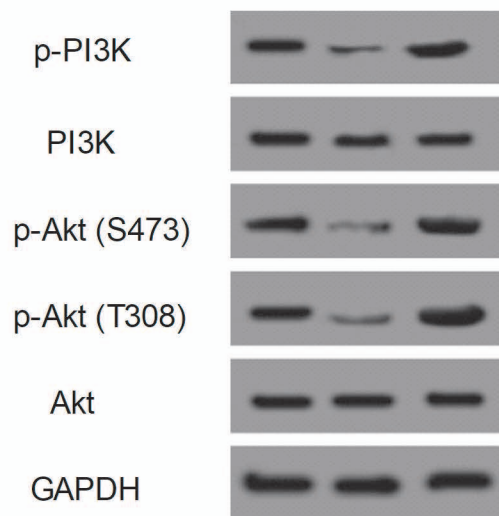**B****786-O**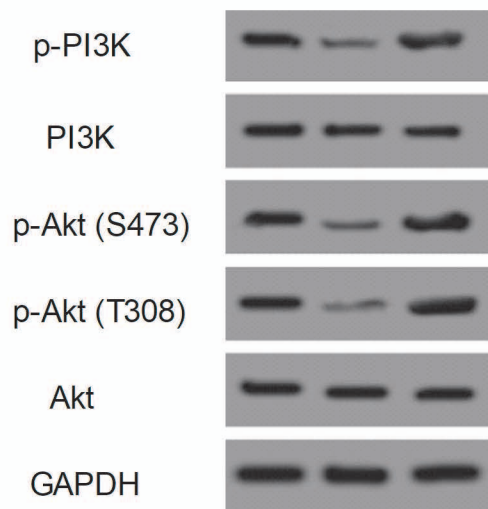

Supplement: Supplementary Materials — Supplementary Fig. 1: PI3K agonist 740Y-P reverses the inhibitory effect of LINC01535 on the PI3K/Akt pathway in ccRCC cells. (A, B) The protein expression of p-PI3K, PI3K, p-Akt (T308 and S473), and Akt in ccRCC cells was measured by western blot following treatment with 740Y-P (20 μmol/L). ∗p < 0.05. [file 2153337.f1.pdf]
